# Preliminary Application of Infrared Thermography to Monitoring of Skin Temperature Asymmetries in Professional Padel Players

**DOI:** 10.3390/s24144534

**Published:** 2024-07-13

**Authors:** Alberto De León-Muñoz, Jose Ignacio Priego-Quesada, Joaquín Martín Marzano-Felisatti, Jose Luis Sanchez-Jimenez, Carlos Sendra-Pérez, Inmaculada Aparicio-Aparicio

**Affiliations:** 1 Research Group in Sports Biomechanics (GIBD), Department of Physical Education and Sports, University of Valencia, 46010 Valencia, Spainj.ignacio.priego@uv.es (J.I.P.-Q.); inmaculada.aparicio@uv.es (I.A.-A.); 2 Research Group in Medical Physics (GIFIME), Department of Physiology, University of Valencia, 46010 Valencia, Spain

**Keywords:** thermal imbalance, thermal images, racquet sports, paddle tennis

## Abstract

The aim of the present study was to evaluate skin temperature (Tsk) asymmetries, using infrared thermography, in professional padel players before (PRE), after (POST) and 10 min after training (POST10), and their relationship with perceptual variables and training characteristics. Thermal images were taken of 10 players before, after and 10 min after a standardized technical training. After training, Tsk of the dominant side was higher than before training in the anterior forearm (30.8 ± 0.4 °C vs. 29.1 ± 1.2 °C, *p* < 0.01; ES = 1.9), anterior shoulder (31.6 ± 0.6 °C vs. 30.9 ± 0.6 °C, *p* < 0.05; ES = 1.0) posterior arm (29.5 ± 1.0 °C vs. 28.3 ± 1.2 °C, *p* < 0.05; ES = 1.0), and posterior forearm (30.8 ± 0.9 °C vs. 29.3 ± 1.6 °C, *p* < 0.05; ES = 1.1). Likewise, these differences were significant POST10 in the anterior arm, anterior forearm, anterior shoulder, posterior arm and posterior forearm. Comparing the different moments of measurement (PRE, POST and POST10), the temperature was higher POST10 in all the regions analyzed except for the shoulder, abdominals, and lower back. Also, correlations were found between fatigue variation and temperature variation between limbs (Tsk dominance), and no correlation was found except between age and posterior thigh (|r| = 0.69; *p* < 0.05), and between the racket mass and anterior knee (|r| = 0.81; *p* < 0.01). In conclusion, infrared thermography allows monitoring of skin asymmetries between limbs in professional padel players, but these asymmetries were not related to overall fatigue variation, overall pain variation, years of experience and training hours.

## 1. Introduction

Padel is a racket sport that has been gaining popularity and scientific interest in recent years, as sport performance and game analysis are among the most investigated topics [1]. In Spain, it is among the 10 most practiced sports [2], and it is expanding, being played in more than 50 countries [1]. Its moderate physical demand, its playful and social nature, and the properties of the court that allow rebounding are some of the characteristics that explain this growth [3].

In padel, the ball is struck with the dominant upper limb, and the repetitive stress of hitting creates muscular imbalances between limbs [4,5], so it is considered an asymmetric sport [6]. In racket sports such as tennis, badminton [6] and padel [7,8], anthropometric differences have been found between both upper limbs. This may lead to anatomical, physical and physiological differences, resulting in an increasing risk of long-term injury to the limb used for ball striking [5,9], and requires preventive interventions to reduce the risk of injury [4,5]. There are different tools for injury detection, being infrared thermography (IRT) an increasingly used technique in sports performance [10,11].

IRT is a non-invasive imaging technique that measures surface temperature (in sports science and medicine, mainly skin temperature (Tsk)), being used for injury detection [12,13], muscle soreness assessment [14], evaluation of the thermal responses to physical exercise stimulus [14], fatigue monitoring during physical exercise [15] or the assessment of thermal asymmetries associated with sports technique [10,16]. Regarding the last application mentioned, the Tsk of humans is symmetrical between both longitudinal sides, and asymmetries greater than 0.5 °C may represent a physiological dysfunction [17]. However, not all asymmetries are related to physiological dysfunctions, and some of them may be associated with adaptations to sport technique execution, such as, for example, in archery [11], handball [18] or tennis [10,19].

Deepening physiological and physical aspects of Tsk behavior during exercise, reference researchers have shown that skin (integumentary system) fundamentally regulates heat exchange by conduction, convection, radiation and evaporation [20,21,22]. Moreover, the increase in core temperature triggers the physiological responses, the sweating and vasodilation [23]. During exercise, changes in skin blood flow occur, related to the individual degree of vasodilation and vasoconstriction [22,24,25]. This is why cutaneous blood flow influences Tsk while exercising [22]. At the beginning of exercise, the demand for blood flow in the active muscles and core causes brief cutaneous vasoconstriction, but as core body temperature increases, thermal regulation processes and the cutaneous vessels dilate, increasing heat dissipation through the skin [21,22,24,26]. In this sense, higher levels of physical fitness are associated with higher skin blood flow, as shown with endurance-trained athletes [26].

To the authors’ knowledge, although IRT interventions were performed in racket sports such as tennis, there are no investigations in padel. Contralateral asymmetries have been found in tennis players, in areas such as the anterior forearm and posterior forearm, as well as a decrease in Tsk in most ROI after training (except posterior forearm, shoulder and leg) [10]. Researchers have concluded that IRT applications could provide relevant information concerning asymmetry control, technical pattern analysis and injury prevention protocols [10,19]. Therefore, this article aims to assess if there are significant baseline Tsk asymmetries in padel players that can be attributed to chronic adaptations or if sport technique asymmetry is revealed in IRF after training, considering the possibility of establishing an IRT protocol for padel players’ injury prevention.

For all the above reasons, the main objective of this work was to analyze the Tsk asymmetries in professional padel players before (PRE), after (POST) and 10 min after the end (POST10) of tactical training and its relationship with the variation (∆)in fatigue and pain, years of experience, racket mass, hours of training and age. Considering a previous study in tennis [10,19], we have hypothesized that in the PRE situation, no differences between both upper limbs would be observed, but after technical training, higher Tsk of the dominant upper limb would appear.

## 2. Materials and Methods

### 2.1. Participants

Ten professional padel players (World Padel Tour and A1 padel) voluntarily participated in this study (9 males and 1 female; age = 22.0 ± 6.0 years, body mass = 73.0 ± 8.8 kg, height = 181.0 ± 9.5 cm, racket mass = 370.0 ± 5 g, body mass index = 22.2 ± 1.8 kg/m^2^, 9.0 ± 4.0 h of paddle per week, 7.0 ± 4.0 years of experience, and 6.0 ± 0.0 days of training per week). The study was conducted under the ethical principles of the Declaration of Helsinki (1964) and approved by the Ethics Committee of the University of Valencia (2626957), and all the participants signed the informed consent form.

### 2.2. Procedures and Experimental Design

**Figure 1:**
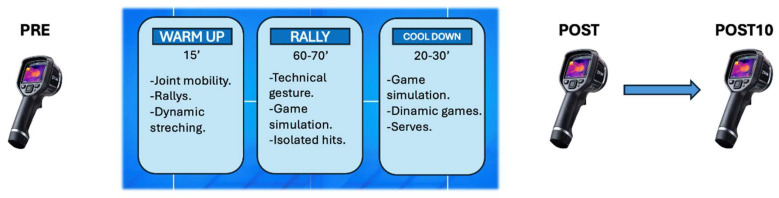
Description of a padel training session and moments of thermographic images acquisition.

The players participated in the same training group, led by the same coach, with a duration on the court of 3 ± 1 h with a common training pattern. The training consisted of a tactical part and game simulations combined with isolated hits (Figure 1). The study was designed with PRE, POST and POST10 measurements 10 min after training (Figure 1). Thermographic images were taken PRE and POST. Overall pain perception was measured using a 15 cm visual analogue scale (VAS) (0 no pain and 15 maximal pain) at PRE and POST moments, calculating variation between POST and PRE (∆Pain) [26]. For the perception of fatigue, the adapted Borg scale (0 no fatigue/10 extreme fatigue) was used [27], and variations were also assessed (∆Fatigue). The scales were shown to the participants, and they pointed to the score selected.

### 2.3. Skin Temperature Assessment

The room temperature and relative humidity were 19.5 ± 1.2 °C and 51.0 ± 3.0%, respectively (TFA Dostmann D-97877 weather station). The IRT camera used was the Flir E60bx (Wilsonville, Oregon, USA) with a sensor array size of 320 × 240 and a noise equivalent temperature difference (NETD) of <50 mK. It was turned on 10 min before measurements for stabilization of the electronics. Thermal images were taken with the players in anatomical position at 2 m distance, with the camera perpendicular to the regions of interest (ROIs). All images (PRE, POST and POST10) were taken under the same fixed range and span conditions for later accurate comparison.

Participants received indications of the study requirements 48 h and 24 h before the test: (i) to train the day of the test with polyester T-shirts and low socks; (ii) not undergo UV treatment 12 h before the test day; (iii) avoid heavy meals the night before; (iv) do not take stimulants or depressants; (v) avoid using therapeutic creams or cold/heat effect sprays; (vi) do not smoke before the test day; and (vii) avoid intense physical exercise 24 h before the test. The thermal adaptation time for the images at PRE was 10 min, and the players maintained the anatomical position during that time [13]. The males were shirtless and the female participant wore sports tops, with their pants pulled up to the thigh and socks pulled down [28].

**Figure 2:**
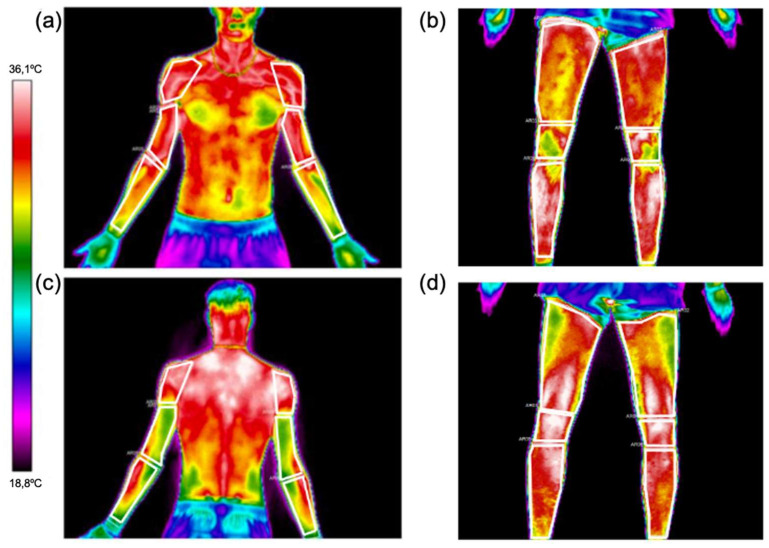
Regions of interest analyzed of (**a**) anterior upper limbs, (**b**) anterior lower limbs, (**c**) posterior upper limb and (**d**) posterior lower limb. Images shown were those of one male professional padel player in the post-exercise measurement moment.

A total of 14 ROIs were selected at the different times analyzed (PRE, POST and POST10) following the Glamorgan protocol on thermal images [29] (Figure 2). In the upper limbs and trunk, the ROIs were anterior shoulder, anterior arm, anterior forearm, abdominals, posterior shoulder, posterior arm, posterior forearm and lower back. In lower limbs, the ROIs were anterior thigh, anterior knee, anterior leg, posterior thigh, posterior knee and posterior leg. ThermaCAM Researcher Pro-2.10 software (FLIR, Wilsonville, OR, USA) was used for the analysis using an emissivity of 0.98 [30]. The variables analyzed for each ROI were mean, maximum, minimum, amplitude (difference between maximum and minimum), and standard deviation. Contralateral thermal asymmetries between dominances (dominant—non-dominant) and variations between POST and PRE (∆Tsk) were calculated. The dominant side was established according to which hitting arm the player used during training.

### 2.4. Statistical Analysis

Statistical analysis was performed using RStudio (version 2023.12.01) and with primary packages (ggstatsplot and corr). As most of the variables followed a normal distribution (*p* > 0.05; Shapiro–Wilk test), data are reported as mean ± standard deviation. Firstly, the dominant and non-dominant comparison was assessed using the Student’s *t*-test for related samples. Secondly, the evolution of Tsk pre-training, post-training and 10 min post-training was assessed by applying a one-way repeated-measures ANOVA, to analyze the differences between moments (i.e., PRE, POST and POST10), and by a pairwise comparison using Student’s *t*-test with Bonferroni correction. Cohen’s effect sizes (ES) were computed to provide information about the magnitude of the differences and were classified as small (ES 0.2–0.4), moderate (ES 0.5–0.7), or large (ES > 0.8) [31]. Finally, to understand the possible factors affecting the skin temperature asymmetries, a correlation bivariate analysis was performed using Pearson’s correlation coefficient to assess the correlation between Tsk dominance (Dominant—Non-dominant) and ∆Fatigue, ∆Pain, years of experience, racket mass, training hours and age. Significant correlations were classified as weak (0.2 < |r| < 0.5), moderate (0.5 < |r| < 0.8), or strong (|r| ≥ 0.8). The significance level was set at *p* < 0.05.

## 3. Results

### 3.1. Dominant vs. Non-Dominant

**Figure 3:**
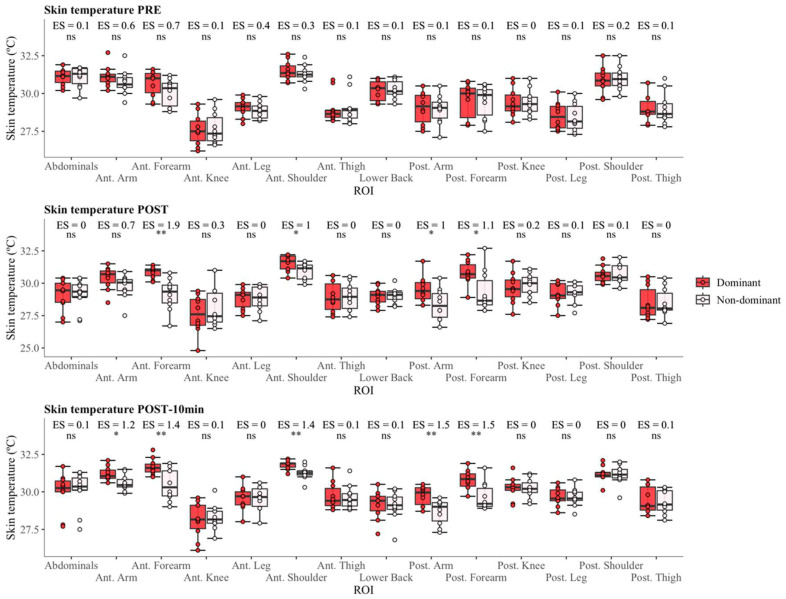
Box plot of differences between dominant and non-dominant sides. Significant differences between sides are identified by symbols (ns, non-significant, * *p* < 0.05, ** *p* < 0.01), with the effect size presented above the symbols.

No differences between both sides were observed at PRE for any ROI (*p* > 0.05, Figure 3). In the POST moment, the differences were in four ROIs: anterior forearm (30.8 ± 0.4 °C vs. 29.1 ± 1.2 °C, *p* < 0.01 and ES = 1.9), anterior shoulder (31.6 ± 0.6 °C vs. 30.9 ± 0.6 °C, *p* < 0.05 and ES = 1.0), posterior arm (29.5 ± 1.0 °C vs. 28.3 ± 1.2 °C, *p* < 0.05 and ES = 1.0), and posterior forearm (30.8 ± 0.9 °C vs. 29.3 ± 1.6 °C, *p* < 0.05 and ES = 1.1). However, at the POST10 moment, differences were found in five ROIs; anterior arm (31.2 ± 0.5 °C vs. 30.6 ± 0.5 °C, *p* < 0.05 and ES = 1.2), anterior forearm (31.7 ± 0.6 °C vs. 30.5 ± 1.1 °C, *p* < 0.01 and ES = 1.4), anterior shoulder (31.8 ± 0.3 °C vs. 31.3 ± 0.5 °C, *p* < 0.01 and ES = 1.4), posterior arm (29.8 ± 0.8 °C vs. 28.7 ± 0.6 °C, *p* < 0.01 and ES = 1.5), and posterior forearm (30.8 ± 0.9 °C vs. 29.7 ± 0.9 °C, *p* < 0.01 and ES = 1.5) (Table 1).

### 3.2. Skin Temperature Variation by Training

**Figure 4:**
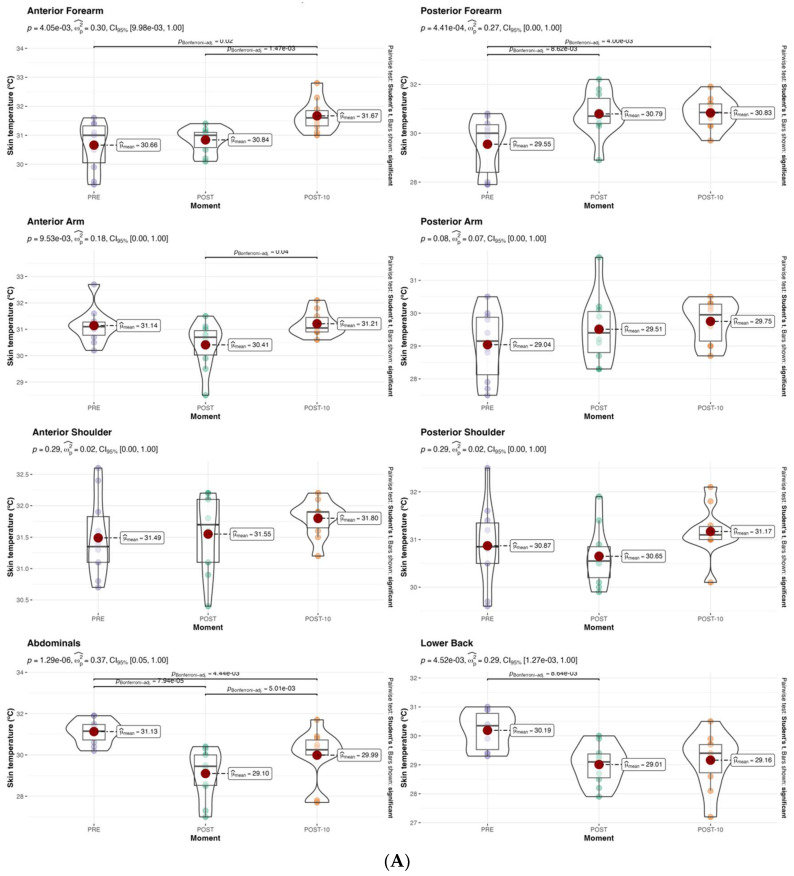

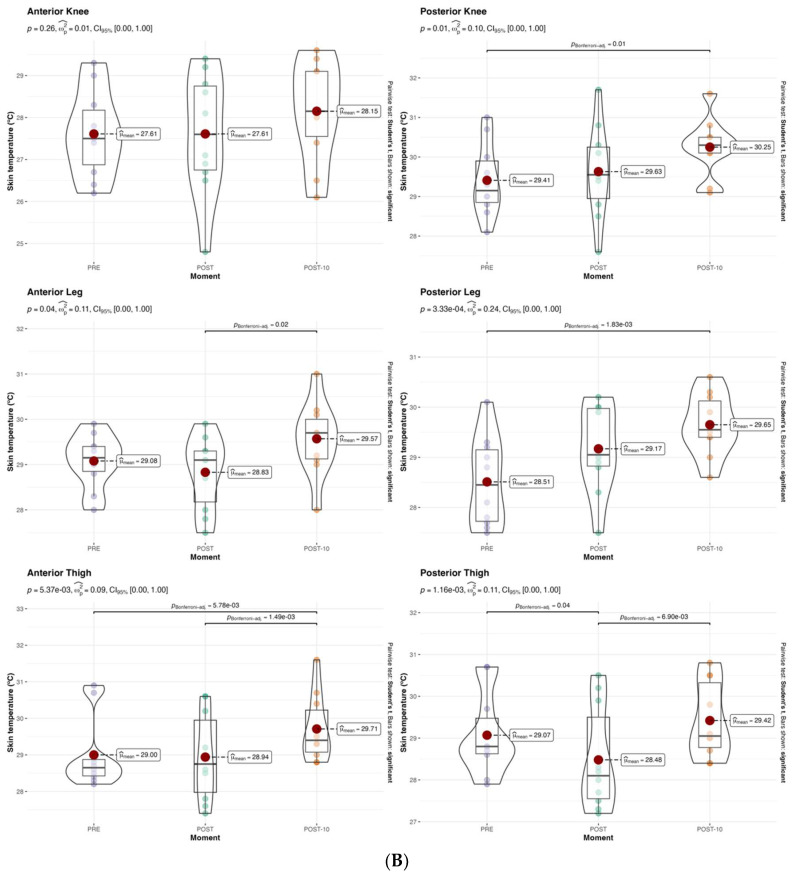
(**A**) Box plot of the differences between moments in the upper body (pre-training, post-training and post-training 10 min). (**B**) Box plot of the differences between moments in lower body (pre-training, post-training and post-training 10 min).

Tsk was higher at POST10 than at the PRE moment in the different regions analyzed, except the shoulder, abdominals, and lower back (*p* > 0.05) (Figure 4A). Regarding upper body, anterior forearm showed differences (PRE vs. POST10 and POST vs. POST10; 30.7 ± 0.8 °C vs. 31.7 ± 0.6 °C, *p* = 0.02 and ES = 1.4, and 30.8 ± 0.4 °C vs. 31.7 ± 0.6 °C, *p* = 0.0014 and ES = 1.7), posterior forearm (PRE vs. POST and PRE vs. POST10; 29.6 ± 1.2 °C vs. 30.8 ± 0.9 °C, *p* = 0.008 and ES = 1.4, 2nd 29.6 ± 1.2 °C vs. 30.8 ± 0.6 °C, *p* =0.004 and ES = 1.4), anterior arm (POST vs. POST10; 30.4 ± 0.8 °C vs. 31.2 ± 0.5 °C, *p* = 0.04 and ES = 1.1), abdominals (PRE vs. POST; 31.1 ± 0.6 °C vs. 29.1 ± 1.2 °C, *p* < 0.001 and ES = 2.1) (PRE vs. POST10; 31.1 ± 0.6 °C vs. 30.0 ± 1.3 °C, *p* = 0.004 and ES = 1.1) and (POST vs. POST10 29.1 ± 1.2 °C vs. 30.0 ± 1.3 °C, *p*= 0.005 and ES = 0.7), and lower back (PRE vs. POST; 30.3 ± 0.7 °C vs. 29 ± 0.7 °C, *p* = 0.005 and ES = 1.8).

In the lower body (Figure 4B), Tsk increased in post-training in all regions: posterior knee (PRE vs. POST10; 29.4 ± 0.9 °C vs. 30.3 ± 0.7 °C, *p* = 0.01 and ES = 1.0), anterior leg (POST vs. POST10; 28.8 ± 0.8 °C vs. 28.2 ± 1.2 °C, *p* = 0.02 and ES = 0.9), posterior leg (PRE vs. POST10; 28.5 ± 0.9 °C vs. 29.7 ± 0.6 °C, *p* = 0.002 and ES = 1.5), anterior thigh (PRE vs. POST10 and POST vs. POST10; 29 ± 1 °C vs. 29.7 ± 0.9 °C *p* = 0.006 and ES = 0.8 and 28.9 ± 1.2 °C vs. 29.7 ± 0.9 °C, *p*= 0.001 and ES = 0.7) and posterior thigh (PRE vs. POST10 and POST vs. POST10; 29.1 ± 1 °C vs. 29.7 ± 0.9 °C *p* = 0.04 and ES = 0.4, and 28.5 ± 1.2 °C vs. 29.7 ± 0.9 °C *p* = 0.007 and ES = 0.9).

### 3.3. Correlations Analysis

The correlation analysis between each Tsk dominance at POST10 (Dominant—Non-dominant) of all regions, and ∆Fatigue (3.5 ± 1.7 cm), ∆Pain (2.7 ± 2.6 cm), years of experience, racket mass, training hours and age are shown in Table 1. Racket mass showed a strong correlation with Tsk dominance in anterior knee (|r| = 0.81; *p* < 0.01). Additionally, player age was moderately correlated with Tsk dominance in posterior thigh (|r| = 0.69; *p* < 0.05) (Table 2).

## 4. Discussion

The aim of the present study was to evaluate the contralateral asymmetries in the Tsk of padel players PRE, POST and POST10 after on-court training and to evaluate the relationships between asymmetries, fatigue, pain, years of experience, racket mass, training hours and age. Additionally, we studied the Tsk variation in PRE training vs. POST and POST10 min after the training session. The main findings showed a greater Tsk in the dominant limb just after training in the anterior forearm, anterior shoulder, posterior arm and posterior forearm, and 10 min after training, these differences are maintained, adding anterior arm. Regarding the evolution of Tsk before and after training, the Tsk was higher 10 min after training in the anterior forearm, posterior forearm and anterior arm of the dominant upper limb and increased in all regions of lower limb. Finally, a strong correlation was found between thermal asymmetries of the knee with racket weight, and a moderate correlation between posterior thigh and age.

### 4.1. Dominant vs. Non-Dominant

Several studies have evaluated thermal asymmetries according to dominances [16,18]; however, few deal with racket sports [10,19]. Our results after padel tennis training show higher temperatures in the hitting arm. The results are in agreement with previous studies on tennis [10,19], archery [11] and handball [18], where athletes showed higher temperatures in their dominant limbs due to the specific characteristics of these sports.

In professional athletes, these contralateral thermal asymmetries are due to the fact that the dominant limbs require greater metabolic demands and, consequently, greater skin vasodilatation after training [32]. Conversely, skin vasoconstriction takes place in muscles with lower demands [11,33]. This resulted in a higher Tsk in muscles with higher metabolic demands [11], in this case, in the dominant limbs. In our findings, it can be seen how the forearms (i.e., anterior and posterior) show significant differences at POST and POST10 (*p* < 0.01 and ES = 1.1–1.9). This may be due to the isometric grip strength that players use for strokes such as volleys, prone–supination movements and the force to hold the racket during training [10]. In addition, there are some ROIs such as the forearm (anterior and posterior), posterior arm and anterior shoulder whose differences exceed 0.5 °C, and special monitoring is required [28].

As is shown in Figure 3, thermal asymmetries remained similar 10 min after the end of training. The musculature that showed greater Tsk differences between sides was the one related to ball striking [34,35], and this increase may be due to accumulated fatigue after training, sport-specific technique or biomechanical patterns of the athlete [28,36]. Similarly, changes in Tsk can vary depending on the sport and its specific technique [11], and Tsk restoration during recovery is prolonged until it reaches baseline values [37].

### 4.2. Skin Temperature Variation by Training

Physical exercise increases metabolic heat production, which results in an increase in muscle temperature, especially in the muscles involved in the activity [12,36]. As shown in Figure 4B, Tsk increased significantly POST10 training in upper limb regions and just after training in all lower limb regions. These findings are not in agreement with previous works [10,38], where, due to cutaneous vasoconstriction and activity-dependent activation of sweating mechanisms [10,39], Tsk was lower after the onset of exercise [12]. This may be due to the intermittent intensity pattern of padel tennis [40,41] or to the protocols carried out by the authors.

In asymmetric sports, the temperature of active regions during physical exercise shows higher temperatures compared to non-active ones [12]. Young professional tennis players show higher temperatures in the anterior and posterior forearm of the dominant arm after technical training (1.1 ± 0.5 °C/1.2 ± 1 °C) [10]. In archery, higher temperature values are observed in the arm supporting the bow [11] and, similarly, in the dominant upper limb of handball players [18]. In these researches, higher Tsk is observed at the end of training due to the technical specificity of the sports [10,11,18], thus coinciding with our results.

### 4.3. Correlation Analysis

There is a strong correlation between the racket mass and the thermal asymmetries of the anterior knee, which can be explained by the player’s movements and position during the game. In a professional padel match, there are 117 ± 19 points (or rallies), and a player hits the ball 9 ± 2 times per game [40,41]. Moreover, during the game, padel players perform specific forward and backward displacements resulting in a positional struggle with the opponent [41]; this explains that padel is a sport where the player is in constant movement with an implement, the racket. During training and matches, the player’s position consists of a semi-flexion of the knees that entails a constant activation of the lower limbs for changes in direction and keeping the center of gravity low [42,43]. On the other hand, previous studies have shown that the mass of the tennis racket affects technical performance and the number of involved muscle fibers of the arm and forearm [10,19,44]. Furthermore, in padel, racket shape affects technical execution and the incidence of injuries [38]. In a complementary way, Marzano et al. (2023) showed that the mass of the racket is correlated with the lower back region [10].

A moderate correlation has been found between thermal asymmetry of the posterior thigh and age. Padel tennis is a sport where repeated accelerations and decelerations take place in a reduced space [4,5]. The posterior thigh musculature is responsible for carrying out accelerations and, above all, decelerations [45], in addition to supporting the body mass (i.e., especially the dominant lower limb) to allow trunk rotation during strikes [46]. In line with the above, older, experienced tennis players show greater capacity for decision making and are able to show higher volume of play and perform more efficiently with more forceful executions [47]. This may translate into greater game responsibility, leading to more strokes and better placement on the court [48,49]. In a complementary way, trained athletes tend to have greater heat transfer capacity due to higher skin blood flow capacity and lower body fat percentage. All these facts may explain the present correlation between age and thermal asymmetry of the posterior thigh.

### 4.4. Experimental Considerations and Future Research

There are some experimental considerations. The sample analyzed was small, and only one female was included, so we should be cautious when correlations are considered. This can be explained by the difficult access to professional players and the competitive moment where the intervention took place. In addition, the training was the same for all players, so the training load cannot be objectively determined. Future research can replicate this study in a larger sample to verify our results. Similarly, it could be interesting to form groups according to sex or age. Furthermore, we recommend increasing the number of variables to make correlations such as the number of strokes per player.

## 5. Conclusions

IRT reveals, in professional padel players, Tsk asymmetries in some of the ROIs assessed between upper limbs in forearm (anterior and posterior), posterior arm and anterior shoulder. These asymmetries increase along the recovery after the training (i.e., 10 min after training), suggesting the presence of a different restoration pattern in Tsk according to the dominant limb. In addition, Tsk dominance in all regions is not related to ∆Fatigue, ∆Pain, and years of experience, although it is related to racket mass in the anterior knee and the age in the posterior thigh. Nonetheless, while these results are preliminary, the use of IRT can provide coaches of asymmetric sports with information on the thermal patterns of their players.

## Figures and Tables

**Table 1:** ROIs with significant differences between dominant and non-dominant sides. ES = Cohen’s effect sizes.

ROIs	Moment	Non-Dominant	Dominant	*p*-Value (ES)
Anterior arm	PRE	30.7 ± 0.8	31.1 ± 0.7	0.23 (0.6)
	POST	29.7 ± 1.0	30.4 ± 0.9	0.12 (0.7)
	POST10	30.6 ± 0.5	31.2 ± 0.5	0.01 (1.3)
Anterior forearm	PRE	30.1 ± 0.9	30.7 ± 0.9	0.15 (0.7)
	POST	29.1 ± 1.2	30.8 ± 0.4	<0.01 (1.9)
	POST10	30.5 ± 1.1	31.7 ± 0.6	<0.01 (1.4)
Anterior shoulder	PRE	31.3 ± 0.6	31.5 ± 0.6	0.47 (0.3)
	POST	30.9 ± 0.7	31.6 ± 0.7	0.04 (1.0)
	POST10	31.3 ± 0.5	31.8 ± 0.3	<0.01 (1.4)
Posterior arm	PRE	28.9 ± 1.1	29.0 ± 1.0	0.83 (0.1)
	POST	28.4 ± 1.2	29.5 ± 1.0	0.03 (1.0)
	POST10	28.7 ± 0.8	29.8 ± 0.6	<0.01 (1.5)
Posterior forearm	PRE	29.5 ± 1.1	29.5 ± 1.2	0.88 (0.1)
	POST	30.8 ± 0.9	29.3 ± 1.6	0.04 (1.1)
	POST10	29.7 ± 0.9	30.8 ± 0.6	<0.01 (1.5)

**Table 2:** Pearson’s correlation coefficient (|r|) between each skin temperature between limbs (Tsk dominance) of regions of interest (ROIs) and overall fatigue variation (∆Fatigue), overall pain variation (∆Pain), years of experience, racket mass, training hours and age. (* *p* < 0.05; ** *p* < 0.01).

ROI (Tsk Dominance)	∆Fatigue	∆Pain	Years of Experience	Racket Mass	Training Hours	Age
	|r|	|r|	|r|	|r|	|r|	|r|
Abdominals	0.	0.08	0.60	0.13	0.01	0.51
Lower back	0.42	0.06	0.16	0.36	0.44	0.36
Ant. Arm	0.12	0.00	0.05	0.31	0.32	0.16
Post. Arm	0.32	0.10	0.18	0.31	0.16	0.09
Ant. Forearm	0.18	0.33	0.12	0.35	0.56	0.01
Post. Forearm	0.33	0.10	0.03	0.13	0.24	0.11
Ant. Shoulder	0.42	0.42	0.32	0.00	0.03	0.14
Post. Shoulder	0.32	0.13	0.16	0.11	0.41	0.21
Ant. Knee	0.00	0.22	0.34	0.81 **	0.49	0.26
Post. Knee	0.18	0.18	0.11	0.32	0.21	0.25
Ant. Leg	0.15	0.05	0.07	0.06	0.34	0.02
Post. Leg	0.03	0.16	0.36	0.02	0.04	0.40
Ant. Thigh	0.42	0.41	0.35	0.04	0.27	0.03
Post. Thigh	0.08	0.12	0.38	0.22	0.38	0.69 *

## Data Availability

The dataset generated and analyzed during the current study is available from the corresponding author upon reasonable request.
